# Extraction Optimization of Water-Extracted Mycelial Polysaccharide from Endophytic Fungus *Fusarium oxysporum* Dzf17 by Response Surface Methodology

**DOI:** 10.3390/ijms13055441

**Published:** 2012-05-04

**Authors:** Peiqin Li, Shiqiong Lu, Tijiang Shan, Yan Mou, Yan Li, Weibo Sun, Ligang Zhou

**Affiliations:** Department of Plant Pathology, College of Agronomy and Biotechnology, China Agricultural University, Beijing 100193, China; E-Mails: lipq110@163.com (P.L.); shiqionglu@126.com (S.L.); shan5400388@163.com (T.S.); muyan01987@163.com (Y.M.); xlfdymmm@yahoo.com.cn (Y.L.); sunweibo.1001@163.com (W.S.)

**Keywords:** optimization, water-extracted mycelial polysaccharide, extraction, endophytic fungus, *Fusarium oxysporum* Dzf17, response surface methodology

## Abstract

Water-extracted mycelial polysaccharide (WPS) from the endophytic fungus *Fusarium oxysporum* Dzf17 isolated from *Dioscorea zingiberensis* was found to be an efficient elicitor to enhance diosgenin accumulation in *D. zingigerensis* cultures, and also demonstrated antioxidant activity. In this study, response surface methodology (RSM) was employed to optimize the extraction process of WPS from *F. oxysporum* Dzf17 using Box-Behnken design (BBD). The ranges of the factors investigated were 1–3 h for extraction time (*X*_1_), 80–100 °C for extraction temperature (*X*_2_), and 20–40 (v/w) for ratio of water volume (mL) to raw material weight (g) (*X*_3_). The experimental data obtained were fitted to a second-order polynomial equation using multiple regression analysis. Statistical analysis showed that the polynomial regression model was in good agreement with the experimental results with the determination coefficient (*R*^2^) of 0.9978. By solving the regression equation and analyzing the response surface contour plots, the extraction parameters were optimized as 1.7 h for extraction time, 95 °C for extraction temperature, 39 (v/w) for ratio of water volume (mL) to raw material weight (g), and with 2 extractions. The maximum value (10.862%) of WPS yield was obtained when the WPS extraction process was conducted under the optimal conditions.

## 1. Introduction

Endophytic fungi refer to those fungi that reside within plant tissues, intercellularly and/or intracellularly, without causing any apparent symptoms of disease [1,2]. During the long period of co-evolution, endophytic fungi play multiple physiological and ecological roles in the mutualistic association with their host plants. For example, many endophytic fungi protect their host plants from the attack of natural enemies such as herbivores and pathogenic microorganisms [3–5], have the special ability to produce the same or similar bioactive compounds originating from their host plants [6,7], and produce the components (e.g. saccharide elicitors) to affect secondary metabolism of the host plants [8–11].

*Fusarium oxysporum* Dzf17 is an endophytic fungus isolated from the rhizomes of *Dioscorea zingiberensis* C. H. Wright (Dioscoreaceae), a well-known traditional Chinese medicinal herb indigenous to the south of China [9,12]. The rhizomes of *D. zingiberensis* have a high content of diosgenin, which is an important precursor of semi-synthetic steroidal drugs in the pharmaceutical industry [13]. In our previous study, water-extracted mycelial polysaccharide (WPS), exopolysaccharide (EPS), and sodium hydroxide-extracted mycelial polysaccharide (SPS) of *F. oxysporum* Dzf17 all showed enhancing effects on diosgenin production in *D. zingiberensis* cell cultures [10]. Among them, WPS was found to be the most effective polysaccharide, which was regarded as a direct or indirect elicitor to regulate growth and diosgenin accumulation [10,11]. Furthermore, WPS was also found to show antioxidant activity [14]. In order to speed up investigations of WPS and subsequent application, a great deal of WPS is needed. However, WPS extracted from *F. oxysporum* Dzf17 mycelia in the previous study was of low yield (*ca.* 5%) [10]. Therefore, it is necessary to optimize the WPS extraction process. To the best of our knowledge, the optimization of the polysaccharide extraction from the cultured mycelia of endophytic fungus *F. oxysporum* Dzf17 has not yet been reported. In this study, Box-Behnken design (BBD) was selected to optimize the extraction parameters by response surface methodology (RSM) [15–18]. Firstly, single-factor experimental designs (*i.e*., extraction time, extraction temperature, ratio of water volume to raw material weight, and number of extractions) were carried out before BBD experiments. Secondly, the more significant factors (*i.e.*, extraction time, extraction temperature, and ratio of water volume to raw material weight), with three levels, were chosen for further extraction optimization of WPS by BBD experiment and RSM analysis.

## 2. Results and Discussion

### 2.1. Effect of Extraction Time on WPS Yield

**Figure 1:**
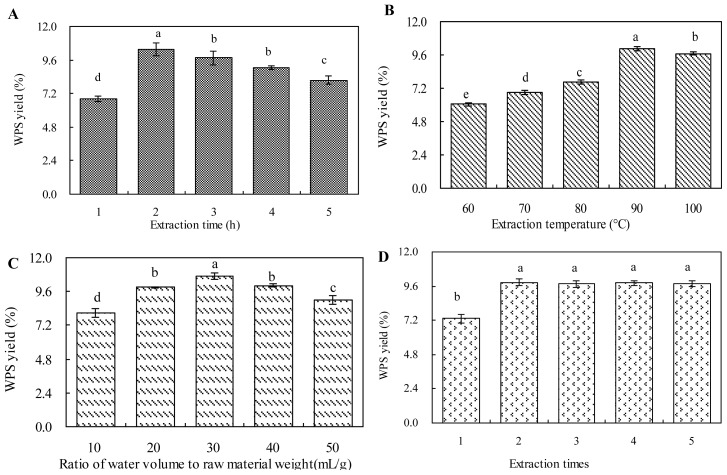
Effects of extraction time (**A**), extraction temperature (**B**), ratio of water volume (mL) to raw material weight (g) (**C**), and number of extractions (**D**) on water-extracted mycelial polysaccharide (WPS) yield. The error bars represent standard deviations from three independent samples. Different letters indicate significant differences among the treatments at *p* = 0.05 level.

Extraction time is an important parameter influencing the extraction efficiency and selectivity of the fluid [19]. It has been reported that a longer extraction time presented a positive effect on the production of polysaccharide [20]. In this study, extraction time was respectively set at 1, 2, 3, 4 and 5 h, while other extraction parameters were fitted as follows: extraction temperature as 90 °C, ratio of water volume (mL) to raw material weight (g) as 30 (v/w), and 2 extractions. The WPS yield increased significantly when the extraction time was prolonged from 1 to 2 h (Figure 1A). WPS yield achieved a maximum value (10.355%) when the extraction time was 2 h. When extraction time was longer than 2 h, WPS yield started to maintain a dynamic equilibrium with a slight decrease. This indicated that an extraction time of 2 h was sufficient to obtain WPS production. A very long extraction time may induce the degradation of polysaccharide, which would lead to changes in the polysaccharide molecular structure and a decrease in polysaccharide production [21]. Therefore, 2 h was selected as the center point of the extraction time in the RSM experiment.

### 2.2. Effect of Extraction Temperature on WPS Yield

Extraction temperature is another factor that plays an important role in the extraction process of polysaccharide. A higher extraction temperature could increase the diffusion coefficient and solubility of the polysaccharide in the extraction solvent [22,23]. To investigate the effect of different temperatures on the extraction yield of WPS, the extraction process was carried out at different extraction temperatures (*i.e.*, 60, 70, 80, 90 and 100 °C), while other extraction parameters were set as follows: extraction time as 2 h, ratio of water volume (mL) to raw material weight (g) as 30 (v/w), and 2 extractions. As shown in Figure 1B, there was an increasing trend in the yield of WPS when the extraction temperature was increased from 60 to 90 °C, and the maximum WPS yield (10.028%) was observed at a temperature of 90 °C. When the extraction temperature was increased from 90 to 100 °C, the yield of WPS decreased slightly. Therefore, 90 °C was selected as the center point of the extraction temperature in the BBD experiment, as a higher temperature would destroy the structure of the polysaccharide and also increase the cost of the extraction process.

### 2.3. Effect of Ratio of Water to Raw Material on WPS Yield

The ratio of water volume to raw material weight would also significantly affect the yield of polysaccharide [24]. In this study, the ratio of water volume (mL) to raw material weight (g) was set at 10, 20, 30, 40 and 50 (v/w) to investigate the influence of different ratios of water volume (mL) to raw material weight (g) on the yield of polysaccharide, while other extraction parameters were fitted as follows: extraction time as 2 h, extraction temperature as 90 °C and 2 extractions. As shown in Figure 1C, WPS yield was significantly increased from 8.052 to 10.704% as the ratio of water volume (mL) to raw material weight (g) was increased from 10 to 30 (v/w), the reason of which was that the increase of ratio of water volume (mL) to raw material weight (g) could enhance the transfer and diffusion of polysaccharide into the extraction solvent [25–27]. However, when the ratio of water volume (mL) to raw material weight (g) continued to increase, WPS yield no longer increased but slightly decreased. Therefore, the ratio of water volume (mL) to raw material weight (g) of 30 (v/w) was selected as the center point in the BBD experiment.

### 2.4. Effect of Extraction Times on WPS Yield

Figure 1D showed the effect of the number of extractions (1–5) on WPS yield while other extraction parameters were fitted as follows: extraction time as 2 h, extraction temperature as 90 °C, and extraction ratio of water volume (mL) to raw material weight (g) as 30 (v/w). As shown in Figure 1D, WPS yield obviously increased when the number of extractions was increased from 1 to 2. With more than 2 extractions, WPS yield no longer changed, which demonstrated that 2 extractions was sufficient for WPS extraction. Thus, 2 extractions were used for the WPS extraction procedure in the succeeding experiments.

### 2.5. Model Building and Statistical Analysis

On the basis of the results of single factor experiments, the three parameters: extraction time (*X*_1_), extraction temperature (*X*_2_) and extraction ratio of water volume to raw material weight (*X*_3_) were chosen as the variables to optimize the process of polysaccharide extraction. There were a total of 17 runs performed for optimizing these three variables in the current Box-Behnken design (BBD) [28]. The values of response *Y* (WPS yield) under the different experimental combinations are presented in Table 1. There was a considerable variation of WPS yield depending upon the different extraction conditions. WPS yield ranged from 7.027 to 10.535%.

The empirical relationships between polysaccharide yield (*Y*) and the tested variables were obtained by application of RSM. By employing multiple regression analysis on the experimental data, the response variable (*Y*) and the tested variables were related by the following second-order polynomial equation (Equation (1)):

(1) $$\begin{array}{l} Y=10.44+0.16\;x_{1}+1.02\;x_{2}+0.36\;x_{3}-0.45\;x_{1}x_{2}-\\ 0.39\;x_{1}x_{3}-0.072\;x_{2}x_{3}-0.70{\;x_{1}}^{2}-1.12{\;x_{2}}^{2}-0.25{\;x_{3}}^{2} \end{array}$$

A summary of the analysis of variance (ANOVA) for the selected quadratic polynomial model is listed in Table 2. The ANOVA of quadratic regression model demonstrated that the model was highly significant, evident from the Fisher’s *F*-test with a very high model *F*-value (345.32) but a very low *p*-value (*p* < 0.0001). The goodness of the model can be checked by the determination coefficients (*R*^2^) and the multiple correlation coefficients (*R*). The value of the determination coefficient adj-*R*^2^ (0.9949) suggested that the total variation of 99.49% for WPS yield was attributed to the independent variables and only about 0.51% of the total variation could not be explained by the model. The value of *R* was closer to 1, the fitness of the model was better [29]. In this research, the value of *R* (0.9989) indicated a high degree of correlation between the observed and predicted values. The lack-of-fit measured the failure of the model to represent the data in the experimental domain at points which were not included in the regression. The *F*-value for the lack-of-fit was not significant (*p* > 0.05), confirming the validity of the model.

The coefficient estimates of model equation, along with the corresponding *p*-values, are presented in Table 3. The *p*-value was employed as a tool to check the significance of each coefficient, which also indicated the interactions between the variables [30]. The smaller the *p*-value, the more significant the corresponding coefficient was [31]. It was concluded from Table 3 that all regression coefficients were highly significant with *p*-values less than 0.01 except for the cross-product coefficient of *x*_2_ (extraction temperature) and *x*_3_ (ratio of water volume to raw material weight).

### 2.6. Response Surface Plot and Contour Plot Analyses

**Figure 2:**
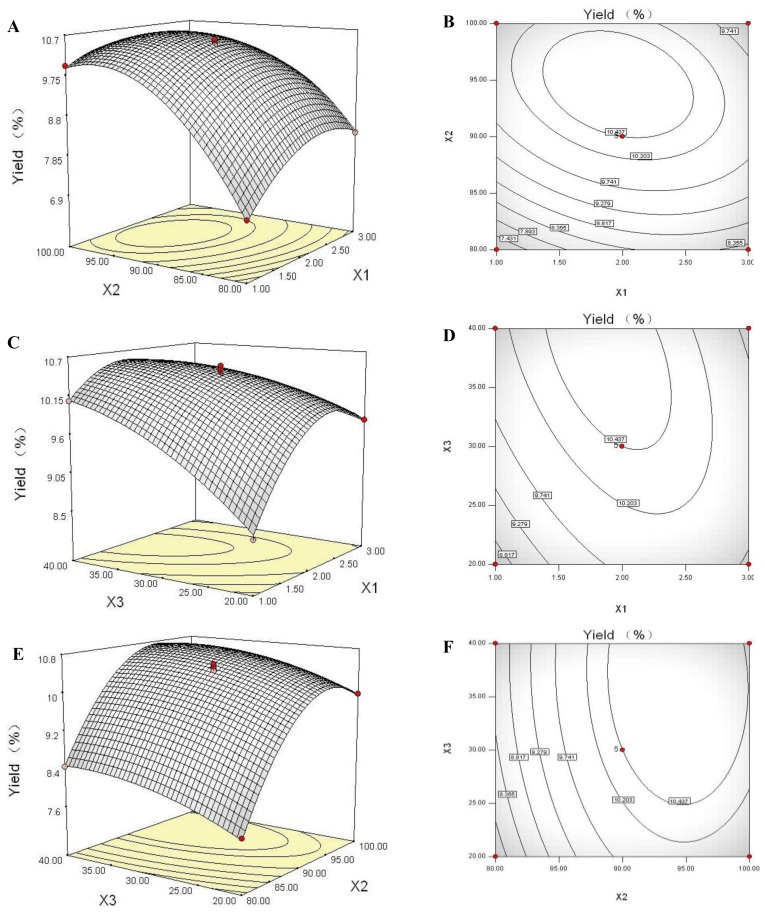
Three-dimensional response surfaces (**A**, **C** and **E**) and contour plots (**B**, **D** and **F**), showing the effects of extraction time (*X*_1_), extraction temperature (*X*_2_), and ratio of water volume (mL) to raw material weight (g) (*X*_3_), and the effect of their their reciprocal interaction on WPS yield (*Y*).

Three-dimensional (3D) response surfaces and two-dimensional (2D) contour plots are the graphical representations of regression equation. They provide a method to visualize the relationship between the response and each variable, and the interactions between any two tested variables. In the present study, the 3D response surfaces and 2D contour plots are presented in Figure 2, which were generated employing the software of Design-Expert 7.1. Analyses of the 3D response surfaces and their respective 2D contour plots allowed us to conveniently investigate the interactions between any two variables, and locate the optimum ranges of the variables efficiently such that the response was maximized. The maximum predicted response was indicated by the surface confined in the smallest ellipse in the contour diagram. An elliptical contour would be obtained when there was an obvious interaction between any two independent variables [31].

The response surface in Figure 2A and contour plot in Figure 2B show the effects of extraction time, extraction temperature and their interactions on WPS yield when the ratio of water volume (mL) to raw material weight (g) was fixed as 30 (v/w). A full elliptic contour was observed in Figure 2B, indicating a significant interaction between extraction time and extraction temperature [31]. The maximum predicted value indicated by the surface was confined in the smallest ellipse in the contour diagram [32]. Thus, the optimal ranges of the two tested variables for obtaining maximum WPS yield were also calculated as follows: extraction time as 1.40–2.39 h, and extraction temperature as 89.92–99.35 °C.

The effects of extraction time and ratio of water volume (mL) to raw material weight (g) on WPS yield at an extraction temperature of 90 °C are illustrated by response surface in Figure 2C and contour plot in Figure 2D. An increase of WPS yield was observed with increasing extraction time at first, but the trend was reversed when the extraction time reached a certain value. In the investigated range of ratio of water volume to raw material weight, WPS yield increased with an increase of ratio of water volume to raw material weight. A similar phenomenon was also observed when the extraction process of the polysaccharide from *Panax japonicus* was optimized by BBD experiment and RSM analysis [33].

Figure 2(E, F) present the effects of extraction temperature and ratio of water volume to raw material weight on WPS yield with an extraction time of 2 h. When the extraction temperature increased from 80 to 95 °C, the WPS yield exhibited a rapid increase, but above 95 °C WPS yield decreased with increasing extraction temperature. At the designed range of ratio of water volume (mL) to raw material weight (g) from 20 to 40 (v/w), an augmentation of WPS production was observed with increasing of ratio of water volume (mL) to raw material weight (g), which is in accordance with the previous studies [34,35].

### 2.7. Optimization of the Extraction Parameters and Validation of the Model

By solving the inverse matrix of the regression polynomial equation employing Design-Expert 7.1, the optimum values of the tested parameters in uncoded units were obtained as follows: extraction time as 1.71 h, extraction temperature as 94.87 °C, and ratio of water volume (mL) to raw material weight (g) as 38.85 (v/w). Under the optimum conditions, the maximum WPS yield was predicted to be 10.822%. Considering the operating convenience of the extraction process, the optimal values of variables were determined as follows: extraction time of 1.7 h, extraction temperature of 95 °C and ratio of water volume (mL) to raw material weight (g) of 39:1 (v/w).

To validate the suitability of the model equation for predicting the optimum response value, experimental rechecking was performed using the deduced optimal conditions. Under the determined conditions, a mean value of WPS yield of 10.862% (*n* = 5) was obtained from the real experiments, slightly higher than the predicted maximum value (10.822%). However, no significant difference was observed between the predicted yield and experimental one when the Student *t*-test was conducted, indicating that the model was satisfactory and adequate for reflecting the expected optimization.

## 3. Experimental Section

### 3.1. Cultivation of Fusarium oxysporum Dzf17

Endophytic fungus *F. oxysporum* Dzf17 was isolated from the healthy rhizomes of *D. zingiberensis* as reported previously [9]. The mycelia of *F. oxysporum* Dzf17 were grown in a 1000 mL Erlenmeryer flask containing liquid medium (300 mL) consisting of glucose (50 g/L), peptone (13 g/L), NaCl (0.6 g/L), K_2_HPO_4_ (0.6 g/L), and MgSO_4_·7H_2_O (0.2 g/L). All flasks were maintained on a rotary shaker at 150 rpm and 25 °C for 14 days. The mycelia were collected by filtration of fermentation broth (150 L), and washed twice with deionized water, then lyophilized.

The dried mycelia (600 g) were powdered in a high power disintegrator, and then subjected to heat circumfluence extraction at 50 °C by 95% ethanol-petroleum ether at 1:1 (v/v) as the refluxing solvent to remove monosaccharide, disaccharide and lipid. The ratio of mycelia powder (g) to refluxing solvent (mL) was 1:5 (w/v). Defatted mycelia powder was obtained by centrifugation (7741 × g, 20 min), and drying in an oven at 40 °C to a constant weight.

### 3.2. Extraction of WPS

WPS extraction was carried out by immersing dried and pretreated mycelial sample (0.5 g for each sample) in hot water at a selected temperature (60–100 °C) for various extraction time (1–5 h) with different ratios of water volume (mL) to material weight (g) from 10:1 to 50:1 (v/w) and different extraction numbers (1–5). The supernatant was collected by centrifugation (7,741 × g, 20 min) and concentrated to a volume of 1 mL. The carbohydrate content was measured spectrophotometrically by the method of anthrone-sulfuric acid [36], which involved sulfuric acid hydrolysis of the sample in the presence of anthrone agent at 100 °C. The absorbance at 620 nm was measured and calibrated to carbohydrate content using glucose as a reference.

### 3.3. Experimental Design

Single-factor experiments were carried out in order to screen out the more significant parameters for polysaccharide extraction, and to determine the proper ranges of these parameters. The factors investigated in this study included extraction time, extraction temperature, ratio of water volume to raw material weight, and extraction number. Based on the results of single-factor experiments, the parameters of extraction time, extraction temperature, and ratio of water volume to raw material weight were chosen for three-factor-three-level Box-Behnken design (BBD) in the analytical process of RSM (software Design-Expert, v.7.1; Stat-Ease, Inc.: Minneapolis, MN, USA, 2007), in order to obtain the optimum extraction conditions for the production of polysaccharide.

Table 4 represented the coded and non-coded values of the experimental variables. The three factors chosen in this study were designated as *x*_1_, *x*_2_, *x*_3_ and prescribed into three levels, coded as +1, 0, −1 for high, intermediate and low, successively. The variable levels *X*_i_ were coded as *x*_i_ according to the following equation (Equation (2)):

(2) $$x_{i}=(X_{i}-X_{0})/\Delta X$$

where *x*_i_ is the coded value of the variable *X*_i_, while *X*_0_ is the value of *X*_i_ at the center point, and Δ*X* is the step change of an independent variable, *i* = 1, 2, 3.

BBD in this experimental design consisted of 17 trials which were carried out in a random order in triplicate that was necessary to estimate the variability of measurements. Five replicates (for the treatments 13–17) at the center of the design were carried out to allow for estimation of a pure error sum of squares. The polysaccharide yield was recorded as the mean of triplicates, which was taken as the response value. Table 1 lists the BBD matrix of the experiment with 17 trials and the response values, which were employed to develop the model.

Based on the experimental data, a second-order polynomial model was established, which correlated the relationship between polysaccharide yield and extraction variables. The relationship could be expressed by the following equation (Equation (3)):

(3) $$Y={\text{a}}_{0}+{\text{a}}_{1}x_{1}+{\text{a}}_{2}x_{2}+{\text{a}}_{3}x_{3}+{\text{a}}_{12}x_{1}x_{2}+{\text{a}}_{13}x_{1}x_{3}+{\text{a}}_{23}x_{2}x_{3}+{\text{a}}_{11}{x_{1}}^{2}+{\text{a}}_{22}{x_{2}}^{2}+{\text{a}}_{33}{x_{3}}^{2}$$

where *Y* is the predicted response value; a_0_ is the intercept term; *x*_1_, *x*_2_ and *x*_3_ are independent variables; a_1,_ a_2_ and a_3_ are linear coefficients; a_12_, a_13_ and a_23_ are cross product coefficients; and a_11_, a_22_ and a_33_ are the quadratic term coefficients. All of the coefficients of the second polynomial model and the responses obtained from the experimental design were subjected to multiple nonlinear regression analyses.

The fitness of the second-order polynomial model equation was evaluated by the coefficient (*R*^2^) of determination. The analysis of variance (ANOVA) and test of significance for regression coefficients were conducted by *F*-test. In order to visualize the relationship between the response values and independent variables, the fitted polynomial equation was separately expressed as 3D response surfaces and 2D contour plots [37,38].

## 4. Conclusions

Based on the single-factor experiments, a Box-Behnken design combined with analysis of response surface methodology was employed to optimize the extraction process of water-extracted mycelial polysaccharide (WPS) of the endophytic fungus *F. oxysporum* Dzf17. The purpose was to determine the extraction parameters that may maximize WPS yield. The maximum value (10.862%) of WPS yield was obtained when the WPS extraction process was conducted under the optimal conditions which were as follows: extraction time as 1.7 h, extraction temperature as 95 °C, ratio of water volume (mL) to raw material weight (g) as 39:1 (v/w), and two extractions. The predicted WPS yield value (10.822%) by the regression polynomial equation showed no significant difference from the experimental yield (10.862%). By optimizing the extraction parameters of WPS from the cultured mycelia of the endophytic fungus *F. oxysporum* Dzf17, WPS yield (10.862%) was about 2-fold higher than that (*ca*. 5%) of the previous extraction [10]. The results should be beneficial for future WPS production from the endophytic fungus *F. oxysporum* Dzf17 as well as for speeding up its research and application.

## Figures and Tables

**Table 1:** Box-Behnken design (BBD) matrix and the response values for water-extracted mycelial polysaccharide (WPS) yield.

Run	*x*_1_	*x*_2_	*x*_3_	WPS Yield (%)		

				Experimental *Y*_e_	Predicted *Y*	*Y*_e_ – *Y*
1	1	−1	0	8.162	8.207	−0.045
2	0	−1	−1	7.649	7.615	0.034
3	−1	−1	0	7.027	6.933	0.094
4	1	1	0	9.318	9.352	−0.034
5	0	1	−1	9.826	9.803	0.023
6	−1	0	−1	8.511	8.579	−0.068
7	1	0	−1	9.695	9.684	0.011
8	1	0	1	9.684	9.616	0.068
9	−1	1	0	9.978	9.933	0.045
10	−1	0	1	10.077	10.088	−0.011
11	0	1	1	10.344	10.378	−0.034
12	0	−1	1	8.456	8.480	−0.024
13	0	0	0	10.437	10.437	0.000
14	0	0	0	10.535	10.437	0.098
15	0	0	0	10.339	10.437	−0.098
16	0	0	0	10.388	10.437	−0.049
17	0	0	0	10.486	10.437	0.049

**Table 2:** Analysis of variance (ANOVA) for the fitted quadratic polynomial model for optimization of WPS yield.

Source	Sum of Squares	d.f.	Mean Square	*F*-Value	Probability (*p*) > *F*
Model	19.24	9	2.14	345.32	<0.0001
Lack of fit	0.019	3	6.44 × 10^−3^	1.07	0.4549
Pure error	0.024	4	6.00 × 10^−3^		
Corrected total	19.28	16			
	*R*^2^ = 0.9978	*R^2^*_adj_ = 0.9949	CV (%) = 0.83		

**Table 3:** Regression coefficient estimates and their significance test of quadratic polynomial model.

Model Term	Coefficient Estimate	Standard Error	Sum of Squares	Mean Square	*F*-Value	Probability (*p*) > *F*
Intercept	10.44	0.035				
*x*_1_	0.16	0.028	0.20	0.20	32.37	0.0007
*x*_2_	1.02	0.028	8.35	8.35	1348.73	<0.0001
*x*_3_	0.36	0.360	1.04	1.04	167.52	<0.0001
*x*_1_*x*_2_	−0.45	0.039	0.81	0.81	130.15	<0.0001
*x*_1_*x*_3_	−0.39	0.039	0.62	0.62	100.45	<0.0001
*x*_2_*x*_3_	−0.07	0.039	0.02	0.02	3.37	0.1089
*x*_1_^2^	−0.70	0.038	2.04	2.04	329.90	<0.0001
*x*_2_^2^	−1.12	0.038	5.28	5.28	852.41	<0.0001
*x*_3_^2^	−0.25	0.038	0.26	0.26	42.14	0.0003

**Table 4:** Coded and uncoded values of the experimental variables.

Variable	Symbol		Coded Level		
		
	Uncoded	Coded	−1	0	1
Extraction time (h)	*X*_1_	*x*_1_	1	2	3
Extraction temperature (°C)	*X*_2_	*x*_2_	80	90	100
Ratio (v/w) of water volume (mL) to material weight (g)	*X*_3_	*x*_3_	20	30	40
